# P-1. Impacts of a Quality Improvement Initiative to Build Confidence in Human Papillomavirus (HPV) Vaccination Among American Indian Communities

**DOI:** 10.1093/ofid/ofae631.212

**Published:** 2025-01-29

**Authors:** Sharon G Humiston, Leah Molloy, Laura Simone, Chris Napolitan, Jeffrey D Carter, Jenniffer A Meza Jimenez, Chelsie Anderson Chadha, Bonnie Douglas

**Affiliations:** Immunize.org, Rochester, New York; PRIME Education, Brighton, Michigan; PRIME Education, LLC, Fort Lauderdale, Florida; PRIME Education, Brighton, Michigan; PRIME Education, LLC, Fort Lauderdale, Florida; PRIME Education, Brighton, Michigan; PRIME Education, LLC, Fort Lauderdale, Florida; PRIME Education, LLC, Fort Lauderdale, Florida

## Abstract

**Background:**

HPV vaccination is highly effective to prevent HPV-associated cancers, but uptake among adolescents and young adults remains suboptimal. This program aimed to identify barriers and support HPV vaccine confidence in American Indian communities in partnership with a Rural Tribal Clinic in Nevada and an urban Federally Qualified Health Center (FQHC) in North Dakota.

**Methods:**

Clinics led 3 live education sessions virtually or in-person from 3/23 to 8/23, which were attended by 91 patients and family members (patients) and 10 healthcare professionals (HCPs). Surveys were administered before, immediately after, and 3 months after each session.

**Results:**

At baseline, 70% of HCPs reported facing hesitancy from patients about the HPV vaccine, with top challenges including patient refusal to discuss vaccination (43%) and sexual stigma around HPV (29%). When asked to identify their patients’ top reason for not getting the HPV vaccine, HCPs reported low perception of self-risk (40%). Conversely, patients reported their top concerns were long-term (39%) and short-term (32%) side effects. When asked what they thought would increase patient interest in vaccination, HCPs identified a strong recommendation from their doctor (60%) while patients prioritized learning more about HPV and associated cancers (57%). Patient HPV vaccine knowledge and confidence improved after sessions. More patients recognized that the HPV vaccine can prevent certain cancers (92% vs 60%, p < .001) and agreed the vaccine is safe (95% vs 64%, p < .001) after sessions than before. Awareness of vaccine access also improved: more patients recognized that vaccines are available free of charge after sessions than before (95% vs 66%, p < .001). Most patients (90%) reported being likely to share what they learned with family and friends, and 100% of providers were likely to lead another session. At 3-month follow-up, HCPs had helped a total of 75 patients get the HPV vaccine.

**Conclusion:**

This initiative highlights the feasibility and impact of clinic-based education sessions on patient vaccine knowledge and confidence and offers insights into community-level barriers and motivators to vaccination.

**Disclosures:**

**Sharon G. Humiston, MD, MPH**, Sanofi: Advisor/Consultant|Sanofi: Honoraria

